# Clinical and Radiographic Benefit of a Patient With Metastatic Non-Small Cell Lung Cancer Harboring an *EGFR::ERBB4* Fusion Through Use of EGFR Tyrosine Kinase Inhibitors

**DOI:** 10.1200/PO-24-00526

**Published:** 2024-12-05

**Authors:** Amy Guimaraes-Young, Kurtis D. Davies, Patricia Trevisan, Hala Nijmeh, Mary Haag, Dara L. Aisner, Tejas Patil

**Affiliations:** ^1^ Department of Pathology, University of Colorado School of Medicine, Aurora, CO; ^2^ Division of Medical Oncology, Department of Medicine, University of Colorado School of Medicine, Aurora, CO

## Introduction

Tyrosine kinase (TK) fusions involving *ALK*, *ROS1*, *RET*, and *NTRK* are known drivers of non–small cell lung cancer (NSCLC), whereas TK domain (TKD) mutations principally mediate *EGFR* tumorigenesis.^1^ Epidermal growth factor receptor (EGFR) belongs to the ERBB receptor family comprising ERBB2 (human epidermal growth factor receptor 2 [HER2]), ERBB3, and ERBB4.^2^ Tyrosine kinase inhibitors (TKIs) have dramatically improved outcomes in patients with TK-driven disease.^3^ Nevertheless, *EGFR* fusions are rare with unclear treatment implications.^4-9^ Here, we describe a patient with *EGFR::ERBB4* fusion–positive NSCLC demonstrating marked response to the EGFR TKI mobocertinib followed by osimertinib.

## Results

### Case

A 58-year-old female never-smoker presented to the emergency department with sudden-onset pleuritic chest pain. She was normotensive and afebrile with 96% oxygen saturation on room air. Physical examination was notable for bilateral bronchial breath sounds. ECG demonstrated no aberrant cardiac rhythms, and D-dimer was within normal limits. Chest x-ray revealed an irregularly marginated lung mass in the right upper lobe characterized by positron emission tomography/computed tomography (PET/CT) as fluorodeoxyglucose (FDG)-avid, 2.0 cm × 1.7 cm, and abutting the anterior pleura. Additional FDG-avid right pleural nodules and hilar, mediastinal, and cervical lymph nodes were also observed. Brain magnetic resonance imaging (MRI) was unremarkable. Right upper lung CT-guided biopsy revealed moderately differentiated adenocarcinoma with immunohistochemical (IHC) staining positive for cytokeratin 7 and thyroid transcription factor and negative for cytokeratin 20, consistent with lung adenocarcinoma. Focused molecular profiling was negative for hotspot *EGFR* or *KRAS* mutations (Sanger sequencing) and *ALK*, *ROS1*, or *RET* rearrangements (fluorescence in situ hybridization [FISH]). The patient was determined to have cT1cN2M1a stage IVA lung adenocarcinoma (American Joint Committee on Cancer 8th edition) with no identified actionable driver alteration. A lung rebiopsy was sent for further molecular profiling, and an *EGFR* intron 27 truncation was reported (Foundation Medicine).

**FIG 1.**
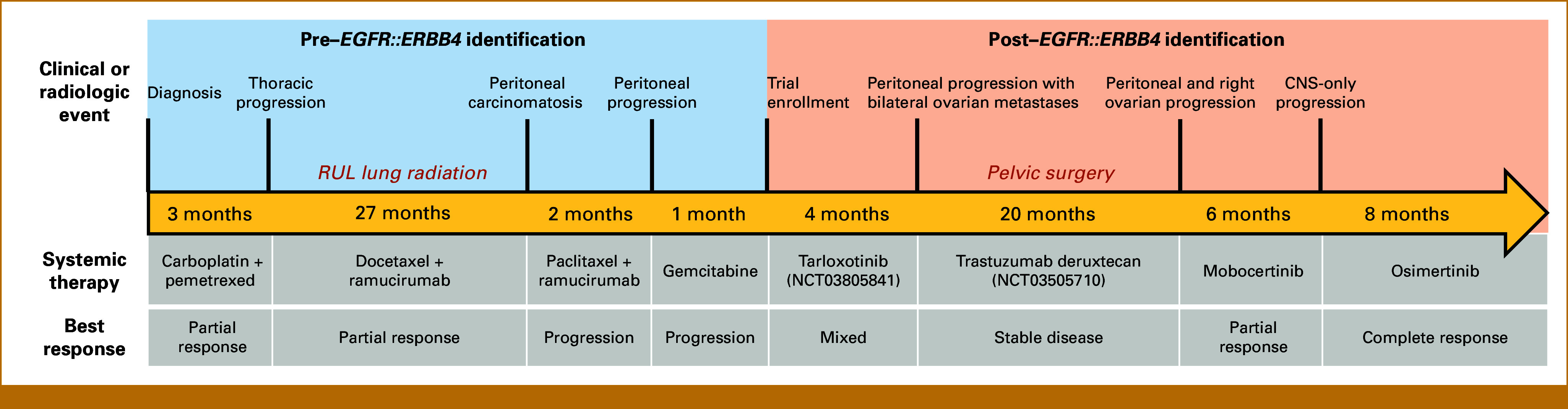
Schematic of the patient's lung cancer treatment course. Durations (in months) of pharmacologic interventions are approximations. Palliative radiotherapy and surgical interventions are annotated above the pharmacologic agent the patient was receiving at the time of procedure. Tarloxotinib and T-Dxd, administered as part of clinical trials, are listed with associated NCT numbers. Radiotherapy administered was SBRT. Pelvic surgery involved laparoscopic lysis of adhesions and left salpingo-oophorectomy. NCT, ClinicalTrials.gov identifier; RUL, right upper lobe; SBRT, stereotactic body radiation therapy; T-Dxd, trastuzumab deruxtecan.

The patient was treated with multiple lines of chemotherapy: (1) carboplatin/pemetrexed, (2) docetaxel 75 mg/m^2^ once every 3 weeks/ramucirumab, (3) paclitaxel/ramucirumab, and (4) gemcitabine. A schematic depicting the patient's treatment course is provided (Fig 1). After the last cycle of gemcitabine, she developed peritoneal progression and was referred to the University of Colorado Cancer Center for clinical trial consideration. At this time, the *EGFR* intron 27 truncation was reclassified as an *EGFR*::*ERBB4* rearrangement (Foundation Medicine). The PD-L1 score was <1%, and tumor mutational burden (TMB) was low (4 muts/Mb).

Given the presence of an *ERBB* family fusion, the patient was enrolled in a phase II clinical trial of tarloxotinib, a hypoxia-activated pan-ERBB/HER kinase inhibitor (ClinicalTrials.gov identifier: NCT03805841). Twenty percent dose reduction was administered after an initial grade 3 infusion–related reaction. On-treatment scans demonstrated mixed response, and tarloxotinib was discontinued after <4 months secondary to further peritoneal progression and development of ovarian metastases.

The patient's tumor was found to have low equivocal HER2 expression (IHC 2+/FISH-negative), and she was enrolled in a phase II clinical trial of fam-trastuzumab deruxtecan-nxki (T-DXd), an antibody-drug conjugate targeting the HER2 receptor (ClinicalTrials.gov identifier: NCT03505710). She had stable disease and remained on therapy approximately 20 months until peritoneal and ovarian progression.

**FIG 2.**
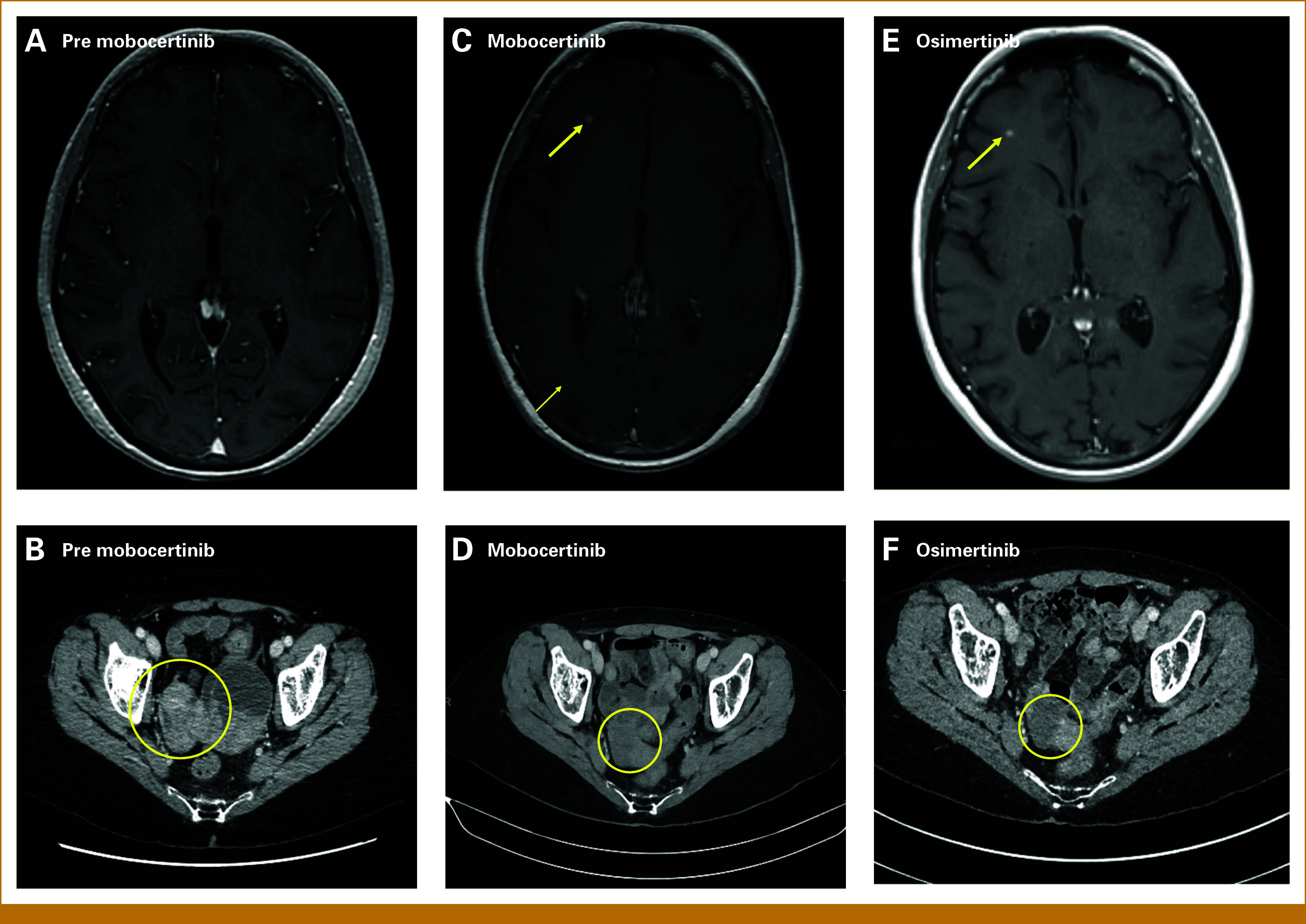
Images demonstrating treatment responses to mobocertinib and osimertinib. (A) Brain MRI 2 months before starting mobocertinib shows no intracranial disease. (B) Chest, abdomen, and pelvis CT before mobocertinib shows an enlarged right ovarian metastasis. (C) Brain MRI after 6 months on mobocertinib shows intracranial progression. Dozens of small, enhancing intraparenchymal foci were detected, predominantly within the supratentorial compartment. Two lesions (yellow arrows) are observed in the image frame. The largest lesion, detected at the gray-white junction of the anterior-inferior right frontal lobe, reached a maximum dimension of 6 mm (thick yellow arrow). (D) PET/CT after 8 weeks on mobocertinib demonstrates a partial response (–62% reduction in largest diameter) within right ovarian metastasis. (E) Brain MRI after 8 weeks on osimertinib shows intracranial response and a residual enhancing focus within the right frontal lobe (thick yellow arrow). (F) Initial chest, abdomen, and pelvis CT on osimertinib shows disease control of the right ovarian metastasis. MRI, magnetic resonance imaging; PET/CT, positron emission tomography/computed tomography.

The patient then received compassionate use mobocertinib, 160 mg once daily PO (per os/by mouth). An EGFR TKI was prescribed in an attempt to target the EGFR portion of the *EGFR*::*ERBB4* fusion product. TKI selection was based on constraints related to the patient's health insurance plan. A brain MRI 2 months before initiating mobocertinib demonstrated no intracranial metastases (Fig 2A). Initial on-treatment PET/CT scan showed complete response of all peritoneal lesions and partial response of ovarian metastases (Figs 2B and 2D). After approximately 6 months, brain MRI revealed dozens of intraparenchymal foci (Fig 2C). Extracranial sites of disease showed ongoing response. Given CNS progression, the patient was switched to osimertinib 80 mg once daily PO, a TKI efficacious against intracranial disease.^10-12^ On-treatment scans showed continuous partial response in the ovaries (Fig 2F). Brain MRI revealed resolution of brain metastases and reduction of a right frontal lobe lesion (Fig 2E). To date, she remains on osimertinib (8 months) with grade 1 paronychia and ongoing disease control.

## Discussion

**FIG 3.**
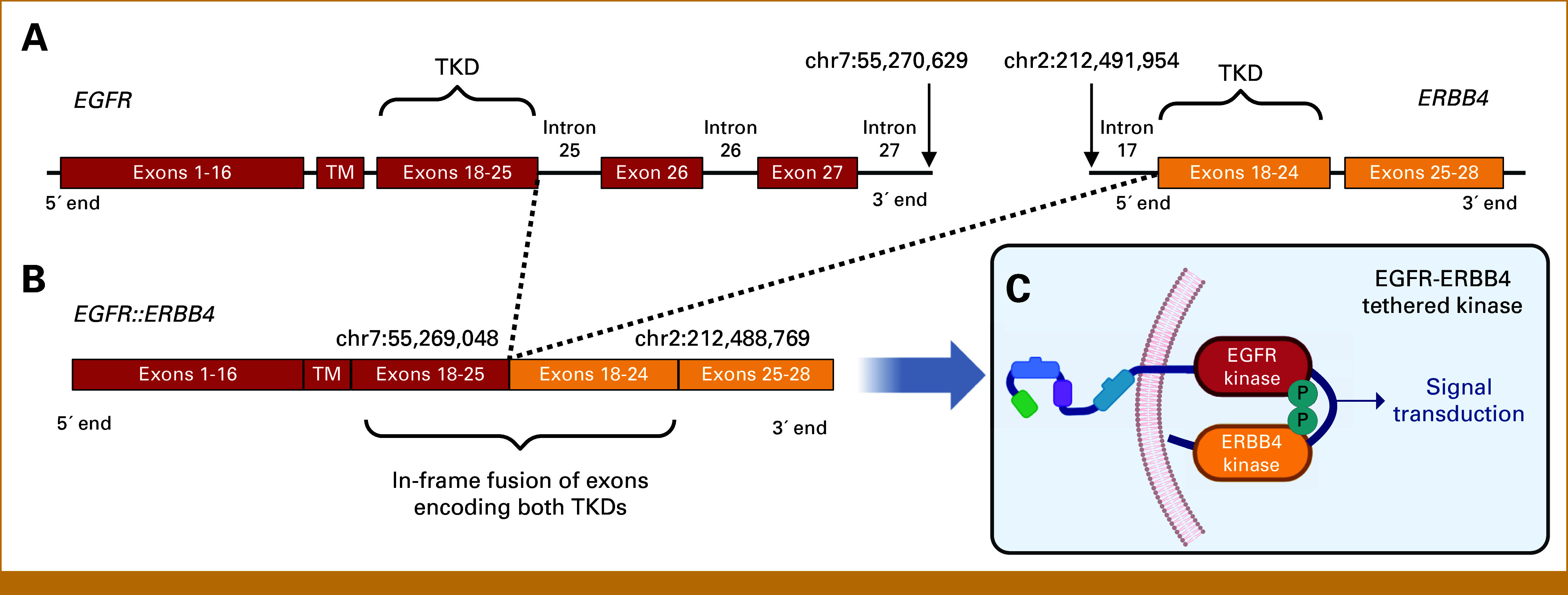
The *EGFR::ERBB4* fusion product leads to juxtaposition of the EGFR and ERBB4 TKDs. (A) The breakpoints of the *EGFR*::*ERBB4* genomic rearrangement as detected by DNA-based solid tumor testing (Foundation Medicine FoundationOne assay) occurred between *EGFR* intron 27 and *ERBB4* intron 17. (B) The breakpoints of the *EGFR*::*ERBB4* fusion transcript occurred between *EGFR* exon 25 and *ERBB4* exon 18, indicating skipping of *EGFR* exons 26 and 27 (Archer FusionPlex). (C) Model of the proposed EGFR-ERBB4 tethered kinase. Formation may lead to constitutive activation and/or altered signal transduction. The figure is designed using BioRender.com. In this artistic rendering, only the introns involved in the exon skipping/splicing event are depicted in the figure. Genomic coordinates of the *EGFR*::*ERBB4* breakpoints (DNA: chr7:55,270,629 and chr2:212,491,954; RNA: chr7:55,269,048 and chr2:212,488,769) are based on the genome assembly GRCh37 (hg19). Exons are based on reference sequences NM_005228.4 (*EGFR*) and NM_005235.2 (*ERBB4*). EFGR, epidermal growth factor receptor; TM, transmembrane-encoded region; TKD, tyrosine kinase domain–encoded region.

To our knowledge, this is the first report of a patient with NSCLC with an *EGFR::ERBB4* fusion responding to TKIs. Initially characterized as an *EGFR* intron 27 truncation by DNA-based testing, it was revised to an *EGFR::ERBB4* rearrangement between *EGFR* intron 27 and *ERBB4* intron 17 (Fig 3A). Subsequent RNA-based fusion testing revealed an in-frame transcript between *EGFR* exon 25 and *ERBB4* exon 18, with skipping of *EGFR* exons 26 and 27 (Fig 3B). Differences between DNA- and RNA-based testing underscore the potential ambiguity of DNA-based characterization and importance of RNA-based testing, which can reveal alternative splicing events.^13^ This fusion product is anticipated to juxtapose the TKDs while conserving ERBB4 protein interaction motifs (UniProt, #Q15303).^14^ The extracellular portion of EGFR is also retained, whereas extracellular and transmembrane portions of ERBB4 are lost. The proposed result is a constitutively active tethered EGFR-ERBB4 kinase (Fig 3C).^15-17^ In publicly available cancer databases, *ERBB4* fusions are rare but recurrent, comprising approximately 0.1% of *ERBB* family fusions, primarily in ovarian, breast, and lung carcinomas.^17^ More rare are *ERBB4* fusions with proposed tethered kinase activation. Indeed, a single *EGFR*::*ERBB4* fusion was reported across databases.^17^ In a study of *EGFR*::*ERBB4*-transduced cells treated with EGFR or pan-HER TKIs including osimertinib and tarloxotinib, levels of activated EGFR, ERBB4, and downstream signaling proteins decreased.^17^ Although EGFR-ERBB4 protein transactivation and oncogenicity were not evaluated, these data suggest that tumors with *EGFR*::*ERBB4* may respond to TKIs.

The reasons for this patient's limited response to tarloxotinib are unclear. Tarloxotinib converts to an active pan-HER TKI (tarloxotinib-E) when exposed to hypoxia, a common state within tumor microenvironments. Proliferation assays have demonstrated tarloxotinib-E–mediated inhibition in the setting of *EGFR* or *ERBB2* exon 20 mutations and *ERBB2* or *ERBB4* amplification.^18^ Wild-type EGFR is also inhibited in hypoxic conditions.^19^ This pan-ERBB activity is predicted to inhibit the *EGFR*::*ERBB4* fusion product. It is possible that altered EGFR/ERBB4 sterics interfered with drug binding. Alternatively, this patient's dose reduction might have rendered subtherapeutic tarloxotinib-E levels.

T-DXd comprises HER2-binding trastuzumab conjugated to a topoisomerase I inhibitor payload that produces a bystander effect on adjacent cells independent of HER2 expression.^20,21^ Growing evidence demonstrates HER2-low (ie, IHC 2+, FISH-negative) tumor response to T-DXd.^22-24^ In the Destiny-Lung01 study, patients with HER2 IHC 2+ or 3+ disease receiving 5.4 mg/kg had a combined objective response rate (ORR) of 34.1%, a disease control rate of 78.0%, and a median duration of response (DoR) of 6.2 months.^25^ Given trastuzumab's high specificity for HER2, this patient's prolonged response to T-DXd is favored to be secondary to HER2-low expression as opposed to the *EGFR*::*ERBB4* fusion.^26^ While speculative, HER2 may also heterodimerize with the fusion product, potentiating T-DXd effects.

The patient had a robust response with mobocertinib, a small-molecule EGFR TKI designed to target *EGFR* and *ERBB2/HER2* exon 20 mutations.^27^ Albeit reduced, mobocertinib also demonstrates inhibitory effects against other kinases including wild-type EGFR, ERBB4, and HER2.^28^ For the purposes of this case report, copy number variation (CNV) analysis of a pretreatment specimen as part of a DNA-based NGS assay confirmed the absence of *EGFR* amplification, recognizing that subtle amplification may be undetectable. Similarly, no *ERBB4* amplification was appreciated. Thus, this patient's response is favored to represent mobocertinib action against the *EGFR::ERBB4* fusion product.

Data from a phase I/II trial evaluating mobocertinib in patients with *EGFR* exon 20–driven disease (ClinicalTrials.gov identifier: NCT02716116) suggest limited intracranial penetration.^29-31^ Patients without baseline brain metastases had an ORR and a median DoR of 56% and 13.8 months, respectively.^29^ By contrast, patients with baseline brain metastases had an ORR and a median DoR of 25% and 5.5 months, respectively.^29^ Moreover, 38% of all patients with progression and 68% with baseline brain metastases demonstrated CNS-first progression.^30^ Our patient's intracranial progression on mobocertinib is consistent with reduced CNS penetrance. Of note, mobocertinib was voluntarily withdrawn from the US market in October 2023 after primary end points of the phase III EXCLAIM-2 trial (ClinicalTrials.gov identifier: NCT04129502) were not achieved.

The EGFR TKI osimertinib is currently first-line therapy for patients with *EGFR* exon 19, exon 20 T790M, and exon 21 L858R NSCLC.^12,32^ Our patient's intracranial response on osimertinib suggests that her progression on mobocertinib reflects limited drug CNS penetrance as opposed to acquired resistance to therapy. Interestingly, osimertinib can inhibit kinases harboring residues analogous to EGFR's cysteine-797 including ERBB4 with its cysteine-803 residue.^33,34^ Thus, it is possible that the patient's response is mediated by inhibition of the ERBB4 kinase in addition to the EGFR kinase.

Benefit from mobocertinib and osimertinib in patients with *EGFR* alterations lacking TKD mutations is intriguing given both drugs' binding affinities for certain TKD mutations.^28,33^ Indeed, patients with NSCLC with other *EGFR* fusions (*EGFR*::*RAD51* and *VOPP1*::*EGFR*) have also demonstrated response to osimertinib.^9,35,36^ Studies using the EGFR kinase domain duplication suggest TKI efficacy secondary to selective inhibition of intramolecular dimer activation.^37,38^ Should the EGFR-ERBB4 tethered protein favor an active EGFR (or ERBB4) kinase, EGFR TKIs may similarly function by inhibiting this active kinase state. Structural modeling of EGFR-ERBB4 may offer more hypotheses as to the mechanism of action of EGFR TKIs against the *EGFR:*:*ERBB4* fusion product.

In summary, to our knowledge, this is the first report of a patient with NSCLC with an *EGFR::ERBB4* fusion demonstrating clinical and radiographic response to EGFR TKIs. These findings suggest a therapeutic option for patients with this rare fusion.

## Methods

### Patient

The patient provided written informed consent for all procedures, tests, treatments, clinical trial participation, and publication of this report. She was consented under an IRB-approved protocol (COMIRB 09-0143).

### Testing

DNA-based NGS rearrangement testing (FoundationOne), PD-L1 IHC (Dako 22C3), and TMB were performed by Foundation Medicine (Cambridge, MA). RNA-based NGS was performed by the Clinical Laboratory Improvement Amendments–certified Colorado Molecular Correlates (CMOCO) Laboratory using a customized Archer FusionPlex Solid Tumor library preparation kit (IDT, Coralville, IA). Libraries were sequenced on the Illumina platform, and data were processed using ArcherDX Analysis package v6.2.3. Indicated exons are based on NM_005228.4 (*EGFR*) and NM_005235.2 (*ERBB4*). CNV plots were generated using CNVkit on a DNA-based custom hybrid-capture NGS assay (CMOCO laboratory).^39^ HER2 IHC was scored per DESTINY-PanTumor02 trial guidelines using Ventana anti-HER2/neu (4B5) monoclonal antibody (Roche Diagnostics, Estes Park, CO).^40,41^ FISH was performed by the Colorado Genetics Laboratory using the PathVysion *HER2* DNA Probe kit (Abbott Molecular). Clinical research approval was obtained, and ethical data use regulations were followed.

## Data Availability

All the data and resources generated for this study are available in the article or from the corresponding author upon request.
